# Towards the bibliography of life

**DOI:** 10.3897/zookeys.150.2167

**Published:** 2011-11-28

**Authors:** David King, David R. Morse, Alistair Willis, Anton Dil

**Affiliations:** 1 Department of Computing, The Open University, Milton Keynes, MK7 6AA, United Kingdom

**Keywords:** bibliography, citation, reference manager

## Abstract

This paper discusses how we intend to take forward the vision of a Bibliography of Life in the ViBRANT project. The underlying principle of the Bibliography is to provide taxonomists and others with a freely accessible bibliography covering the whole of life. Such a bibliography has been achieved for specific study areas within taxonomy, but not for “life” as a whole.

The creation of such a comprehensive tool has been hindered by various social and technical issues. The social concerns focus on the willingness of users to contribute to the Bibliography. The technical concerns relate to the architecture required to deliver the Bibliography. These issues are discussed in the paper and approaches to addressing them within the ViBRANT project are described, to demonstrate how we can now seriously consider building a Bibliography of Life. We are particularly interested in the potential of the resulting tool to improve the quality of bibliographic references. Through analysing the large number of references in the Bibliography we will be able to add metadata by resolving known issues such as geographical name variations. This should result in a tool that will assist taxonomists in two ways. Firstly, it will be easier for them to discover relevant literature, especially pre-digital literature; and secondly, it will be easier for them to identify the canonical form for a citation

The paper also covers related issues relevant to building the tool in ViBRANT, including implementation and copyright, with suggestions as to how we could address them.

## What is a Bibliography of Life?

At the time of writing, the first result when searching for “Bibliography of Life” is Rod Page’s blog post from October 2010, *Mendeley, BHL and the “Bibliography of Life”* (Page 2010). In his post, Rod offers this definition:

“bibliography of life,” a freely accessible bibliography of every taxonomic paper ever published.

The principle of *freely accessible bibliographies* already exists in taxonomy, albeit focused in particular domains, such as ants (e.g., Antbase, http://antbase.org/) or fish (e.g., Fishbase, http://www.fishbase.org/). The aim of the Bibliography of Life is to employ the same approach as these existing bibliographies, but on a far more ambitious scale. The domain covered by this bibliography is to be the whole of taxonomy.

There is a precedent for this ambition. In the domain of Computer Science, the *Digital Bibliography & Library Project* (DBLP, http://www.informatik.uni-trier.de/~ley/db/) evolved from a small specialized bibliography to a digital library covering most subdomains of computer science (Ley 2009). The increase in scope was driven by the library’s users. From small beginnings, the bibliography now lists more than 1,700,000 publications (as at September 2011). At a larger scale and in a different discipline, biomedical science, PubMed (http://www.ncbi.nlm.nih.gov/pubmed) is a well-known database that provides free access to the MEDLINE database of references and abstracts. Both of these databases are maintained by publicly funded institutions rather than commercial organisations. The DBLP is hosted by the Universität Trier, in Germany and the PubMed database is maintained by the United States National Library of Medicine (NLM, http://dtd.nlm.nih.gov).

There is a similar drive in taxonomy to produce a comprehensive library and matching bibliography. We do not see commercial organisations rising to this challenge. For while there are excellent resources, such as Thomson Reuters’ BIOSIS (http://thomsonreuters.com/products_services/science/science_products/a-z/biosis/), the focus in extending these resources is generally on modern, born-digital material, which is both relatively easy to process and potentially commercially profitable through copyright access charges. Taxonomic research is informed by the full history of publications in the subject, and so compared to many other sciences, the historical taxonomic literature remains relevant to current research. In general, commercial organisations do not appear to be actively extending their coverage of the historic literature. Hence, a number of digitisation projects exist, such as the Biodiversity Heritage Library (BHL, http://www.biodiversitylibrary.org/), that attempt to bring old paper documents into the digital age. There remains the problem, however, of producing a comprehensive bibliography of the newly digitised documents. We suggest that while the concept of a *bibliography of life* might be easy to define, the simple fact that it does not exist indicates there are practical difficulties with the idea. This article explores some of these difficulties, and a possible solution.

## Creating the Bibliography of Life

**Figure 1.**
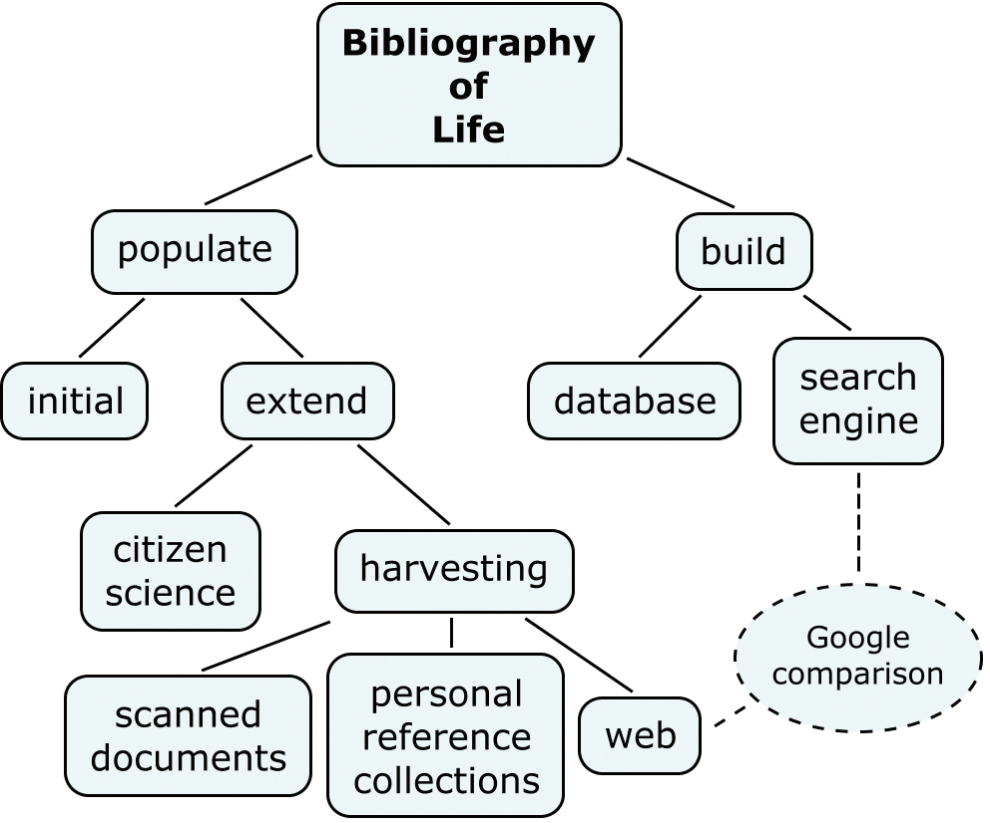
Social and Technical Aspects of the Bibliography of Life

There are two aspects to the creation of the Bibliography of Life. The first is the social aspect, which involves collecting the references and the second is the technical aspect, which involves providing the infrastructure to hold the references. The two aspects are shown in Figure 1 as *populate* and *build* respectively. Other boxes in the figures show how the issues discussed in the this paper relate to these two aspects that are involved in creating the Bibliography of Life.

We intend to *populate *the bibliography with references in two stages. There is the *initial* load from currently available sources to achieve critical mass and to prove the infrastructure. For sustainability we will provide the ability to *extend* the reference collection in the Bibliography of Life. This will be achieved by *harvesting* more resources, including those not generally accessible such as *scanned documents* and *personal reference collections*, and by harvesting *web* hosted resources including Scratchpads. This will be augmented by contributions through *citizen science*, such as the manual addition of references, as well as enabling all users to edit and refine references.

To support the Bibliography of Life infrastructure we intend to build two components. A *database* to hold the references, and a *search engine* to exploit the rich data available to us through holding our own copy of the references, including our own keyword lists and cross-links to original documents.

Hence, the bibliography of life will provide more support for the working taxonomist than existing webbased search engines, such as Google or Google Scholar. Figure 1 shows the points of comparison between a web-based search engine and the proposed Bibliography; in addition to data that can be harvested from the web, the Bibliography of Life must also harvest scanned documents and personal reference collections. The Bibliography will also provide its own database system that stores the key taxonomic facts and allows search to be optimised across these. The rest of this paper considers these steps towards delivering the Bibliography of Life in more detail.

## Loading the initial set of references

The initial set of references for the Bibliography of Life’s can be gleaned from existing resources. Biostor (http://biostor.org/) has demonstrated that a number of references – 63,873 as at November 2011 – can be accumulated relatively easily. However, this number is still relatively small. There has been some discussion (Hull 2010) around the notion that there are some fifty million published journal articles alone. Though this number covers all domains, it does suggest the scale of task in building a comprehensive Bibliography.

Owing to funding patterns there are many smaller bibliographic resources available to provide the initial set of references for the Bibliography of Life. In general, funding is predicated on breaking a big problem into smaller, manageable chunks. In consequence, there has been a multiplicity of databases built. In the absence of large-scale funding a *cottage industry* approach has taken hold, with those researchers interested in the technology and problems of bibliographic reference management building systems in their own personal time. This has meant that opportunities for added value are often missed, while large-scale challenges such as de-duplication and automatic validation are not addressed. The resulting resources are useful, but limited in their scope. They are, however, available for harvesting to populate the Bibliography of Life.

There are a variety of tools we can exploit or extend to harvest references. One such specifically designed for the taxonomic domain is FaLX, developed as part of the European Distributed Institute of Taxonomy project (EDIT, http://www.etaxonomy.eu/). It could aggregate references from Connotea (http://www.connotea.org/), Scratchpads and CiteULike (http://www.citeulike.org/). We have not yet determined which harvesting tool will best serve our needs, or if we will need to develop our own.

## The added value of a large-scale tool

This section discusses the added value we seek to achieve with the creation of a largescale Bibliography. We intend it to represent something more than the sum of the content of existing, specialist bibliographic resources.

### De-duplication

Ideally each target article should have a unique reference. However, multiple references can arise from the import of the same accurate reference into a bibliography from different sources, and also by the existence of near identical references to the same document. How to reduce duplication of bibliographic references remains an open problem in digital libraries research (Kan and Tan 2008). When a search retrieves many identical references to the same article the duplicates are easily ignored and only one copy of the reference is retained. A good cue for this is to check the Digital Object Identifier (DOI http://www.doi.org/) first. However, even if the DOI is the same, sometimes other data can be contradictory or incomplete. It is resolving these *near* identical references that can be difficult. A variety of resolution techniques are required because the problems can come from a variety of sources, such as using different journal abbreviations or a mismatch between fascicle and article page numbers.

The problem of reference de-duplication in bibliographic databases is more formally known as ***citation matching*** (Lee et al. 2007, Kan and Tan 2008), and improving on existing techniques will form one of the core areas of research for Work Package 7 in the ViBRANT project. A preliminary review of the landscape suggests that deduplication techniques developed in information extraction and database management, and applied in other domains are not yet widely used in digital library curation. For example, we have found examples of citation tools being used to detect plagiarism (Plagiarism Today 2011), which might have transferable techniques we can exploit.

### Internationalisation

Internationalisation is a common cause of near identical matches. This can occur when there are multiple names for the same entity such as place names or person names. Also problems arise with the transliteration of entities into Latin script. A topical example is that of the name “Gaddafi”, which is also frequently transcribed as “Kadafi” or “Qaddafi”. There are many variations of the name in Latin script, a problem compounded by the choice of formal Arabic pronunciation of the name or the Libyan dialect, and whether the name is transliterated for an English or French speaking audience (Time:Gaddafi 2011). Even equipped with this knowledge, however, no consensus has emerged on a unique Latin rendering (Yahoo:Gaddafi 2011).

The personal name problem is compounded by cultural differences, affecting such characteristics as name order. This can give rise to further variations depending on whether the name order is amended to match the typical Western style of given name first when the name is transliterated. The World Wide Web Consortium (W3C, www.w3.org/) has produced advice on handling this aspect of internationalisation (W3C:personal names) and other aspects of internationalisation too (W3C:internationalisation). Personal name variations are currently addressed by a variety of techniques including data mining (Phua et al. 2006), while Biostor implements Feitelson’s (Feitelson 2004) weighted clique algorithm for finding equivalent names. These techniques achieve at most 85–90% accuracy, so there is room for further improvement in addressing this difficult problem. In addition, automatic matching techniques do not allow for the occasions when a researcher may deliberately use a different name for different publications, such as to distance themselves from their early work (McKay et al. 2010). As we can expect to encounter variations in author names stored in the Bibliography of Life, we expect to complement the automatic resolution services with an internal look up table to reconcile variations in the spelling of author names. This look-up table could be provided as a separate resource that could be queried via a web service.

Geographical names constitute a similar problem for the Bibliography of Life. For example, Lusaka, the capital of Zambia has been known in the past as Lusaaka, Lusaakas, Lusakas, Lusaka’s and Lusaaka’s. The general problem is compounded by the fact that spellings tend to be less codified in older sources.

Similarly, in the authors’ previous work on the ABLE project (Automatic Biodiversity Literature Enhancement, http://able.myspecies.info/) we encountered an issue with the Anglicised spelling of central American locations in the *Biologia CentraliAmericana*: there was a consistent pattern of replacing an ‘i’ with a ‘y’. Successful data mining of the literature identified by the Bibliography could allow us to build another look up table to help taxonomists resolve these name differences.

### Journal abbreviations

A second common cause of mismatches is the varied abbreviations of journal names. Modern titles tend to follow the ISO 4 standard for abbreviating words and draw on the words in the ISSN’s “List of Title Word Abbreviations” (http://www.issn.org/2-22660-LTWA.php). However, this does not apply to historic literature, with references to titles abbreviated before the international standard was codified. Similar techniques to resolving personal name variations can be applied to journal abbreviations. This collated list of variations could also be provided as a separate resource, which could be queried via a web look-up service.

### Data quality

The question of data quality is not a new one, and it has many dimensions such as completeness, accuracy, correctness, currency and consistency of data (Redman 1996). Data quality can arise whether the reference is user submitted or harvested from an on-line library. There is no guarantee in either case that the input is validated. It would be a disservice to its users if the Bibliography of Life permitted the propagation of bad data.

Manual validation of the data is possible, and a Bibliography of Life requires an editing facility so that users can amend references. Such a service will be developed in ViBRANT by extending the functionality of the GoldenGATE editor so that it can commit the changes back into the Bibliography of Life. However, care must be taken by users editing bibliographic details since this could allow the introduction of new errors, typically through miskeying the intended change.

For the automatic addressing of quality issues, Ley and Reuther (2006) suggest two broad approaches.

The first approach to data validation they call *database bashing*. In this approach the data are checked against other databases. Unfortunately, this is not a foolproof approach because it is possible that both databases contain wrong data derived from a common source, and so an error can be propagated without detection. However, we will, where possible, check against external databases, although it is our ultimate goal that the Bibliography of Life will itself become the authoritative database for taxonomic references.

The second approach to data validation suggested by Ley and Reuther (2006) is *data edits*. This is the application of rules to highlight/resolve discrepancies. This can help address issues such as the Hungarian and Japanese use of family name first when giving names, which may or may not be amended to given name first in the reference. This approach is clearly limited to addressing known issues and common mistakes made when citing references.

We will use both approaches: referring to external resources and applying rule based corrections, to enhance data quality.

Thus far in the Bibliography of Life we have taken existing data and applied some initial steps to ensure the quality of the data. However, this alone will not ensure that the Bibliography of Life is a success.

## Sustainability: extending the set of references

It is necessary that the Bibliography of Life adds sufficient value to working taxonomists so that they continue to engage with it. This is the critical success factor we see in delivering the Bibliography of Life. The initial set of references is unlikely to achieve this, despite the advantages of data quality and quantity that it offers compared to smaller, more specific reference databases. We have the social challenge of building a community of users for whom it is worth their time and effort to contribute to the Bibliography of Life. This problem is potentially self-resolving once there are enough users and enough references to make it a truly useful resource. The question, of course, is how to achieve that desirable critical mass?

This is where building the Bibliography of Life through a larger project such as ViBRANT will be crucial, for ViBRANT gives users another reason to engage with the environment in which the Bibliography of Life is hosted.

## How the Bibliography of Life would be used

**Figure 2.**
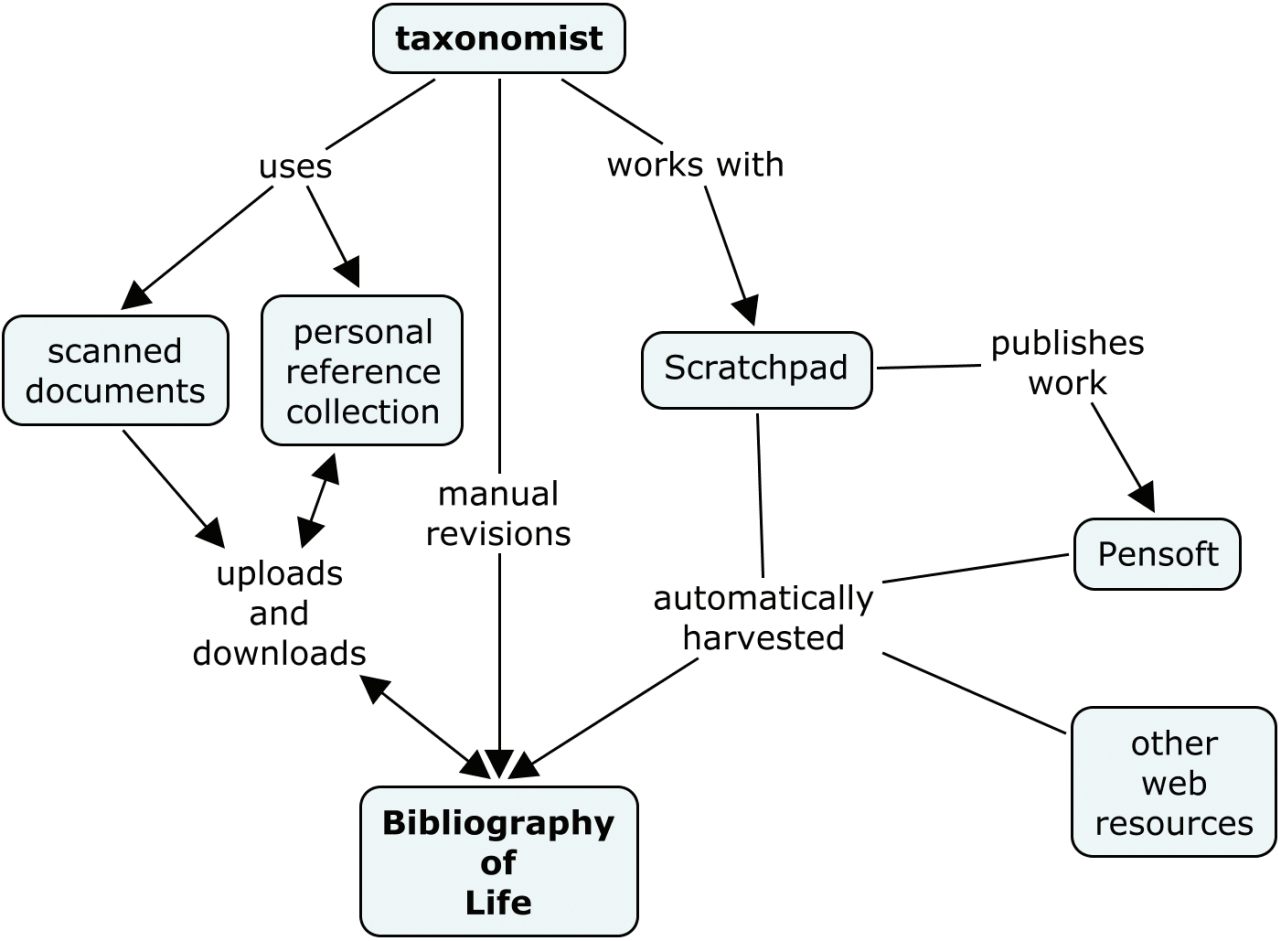
Interactions between a taxonomist and the Bibliography of Life

We recognise that for the successful uptake of the Bibliography, it must integrate easily into the taxonomist’s daily workflow. If interacting with the Bibliography becomes an onerous additional task, then the Bibliography will not be used. A possible workflow is shown in Figure 2.

The ViBRANT environment provides Scratchpads (http://scratchpads.eu/), an on-line tool for taxonomists, encompassing open science and open publication in conjunction with social networking. The feature of Scratchpads most relevant to the Bibliography is the ability for users to store and share their bibliographies. Potentially then, Scratchpads will provide an important resource for a Bibliography of Life. To make this as simple as possible for Scratchpad users, references entered into their Scratchpad will be automatically validated against the Bibliography of Life and added to it, if necessary. In addition, new material published through Scratchpads and ViBRANT partner Pensoft (http://www.pensoft.net/) will automatically be added (Figure 2).

Complementing these two sources of new references, we will continue to revisit periodically the specialist databases used to provide the initial set of references. This will be supplemented by an extended web harvester, to access other less specialised web-hosted resources that contain relevant data. We can endeavour to test our coverage against that of generic search tools such as Google, so that there are not major gaps in our coverage of readily accessible references. There is, however, yet another source of smaller academic databases we wish to access.

Researchers maintain personal databases of domain relevant academic literature. These may be in formal personal reference management tools or simply as *ad hoc* Word documents. We intend that the Bibliography will accept data in all the common bibliographic reference styles, such as BibTeX and Endnote, as well as text strings in, for example, Word documents. To ensure that the Bibliography of Life is relevant to our users we will also have to provide matching export formats.

A related source of data is to parse literature directly for references, such as that held by the individual taxonomist. Parsing literature is a difficult problem, even for major commercial concerns such as Mendeley (Mendeley:reference extraction 2010). One simple technique is to look for the isolated word "References" in the body of the text and examine the subsequent text. This is one of the methods used by open source tools such as ParaCite (http://paracite.eprints.org/) and can be effective on born-digital literature and on well-scanned historic literature. However, as a technique such keyword searches are limited in scope and depend on references being in a dedicated section within a document. Greater problems of automated extraction are provided by embedded references or, worse still, references in an endnote or footnote. Research into reference extraction from across the wide variety of historic taxonomic literature is one of our research goals within ViBRANT.

A further source of references, but one which brings another set of complications, are micro-citations. This is the minimal citation style peculiar to taxonomy, used by nomenclators. By their incomplete nature, satisfactorily resolving the citation is difficult (Gupta et al. 2009) though there are some examples we can build on to address the issues (Page 2011b). If the Bibliography of Life is to be the comprehensive tool envisaged, then we will need to incorporate micro-citation capture. This too, is the subject of one of our research goals.

Automatic extraction can be complemented by supported user input, as exemplified by GoldenGATE (http://plazi.org/?q=GoldenGATE), in which the user first identifies the reference which can then be parsed by the software extraction routines (Sautter et al. 2007). This is a useful facility for a user to add references as they read and review a document. This facility will be available to the Bibliography of Life.

### Using other people’s data: the issue of copyright

“Could it be true that laws designed more than three centuries ago, with the express purpose of creating economic incentives for innovation by protecting creators’ rights, are today obstructing innovation and economic growth? The short answer is: yes.” (Hargreaves 2011)

For the Bibliography of Life, copyright is an issue because current law prevents automated text processing for purposes such as harvesting texts for references. Although it is possible to negotiate a licence to do such processing with the rights holder (usually the academic publisher) on a case by case basis, this is impractical in general, and impossible in the case of orphan works, where the copyright holder is not known.

Some organisations choose to avoid working with potentially copyrighted materials simply to avoid the risk of copyright infringement. In our domain, BHL generally follows this approach, though working with information aggregators such as BioOne (http://www.bioone.org/) has enabled BHL to expand access to more recent, copyrighted publications (Rinaldo and Norton 2010). However, we do not have the option to ignore copyrighted material if we are to build a truly comprehensive Bibliography of Life that includes the modern literature.

Swiss-based Plazi (http://plazi.org/) have used the copyright laws particular to Switzerland to automatically extract taxonomic information from texts. However, these laws do not apply outside the Swiss jurisdiction and in any case, Plazi also argue (Agosti and Egloff 2009) that a system based on legal licensing is more desirable.

Without a resolution to this problem of licensing, the Bibliography of Life might be left with a gap in its records that undermines its sustainability. However, the Bibliography is not intended solely for the professional taxonomist. In other target user groups, some of the problems identified above may not arise.

The Bibliography is not intended solely for the professional taxonomist. In another target user group we may be able to circumvent some of the problems identified above.

### Not just for professional taxonomists

The Bibliography of Life could also facilitate the work of citizen scientists. We expect such individuals to be competent taxonomists, being, for example, retired professional researchers or highly motivated amateurs. We do not envisage a role for more casual citizen scientists such as secondary school students in using and managing bibliographic references. We anticipate that citizen scientists will interact with the Bibliography of Life in a similar manner to the professional taxonomist. However, they will not have the same access to other professional tools so we must ensure that the Bibliography can adapt to their more *ad hoc* use of it. Following the lead of other domains of research, we hope that the citizen scientist will be particularly helpful with quality control by manually reviewing ambiguous data and by engaging in other manual processing of documents to, for example, identify taxon names. We will need to cooperate with the outreach partners within ViBRANT to encourage this behaviour in our users.

Underpinning this expected use of the Bibliography is the technical infrastructure to deliver it.

## How to build it

There are two possible archse an existing database. Building our own database gives us complete control over what we build so we can tailor it to meet our users’ needs. While the first option sounds desirable, it does have to be built and carries the risk, through being yet another tool, of not achieving a critical mass of users.

The alternative is to build on another’s database, leaving us only to ensure the sustainability of our taxonomic specific software enhancements. Of the currently available storage solutions, there are three front runners, in the commercial sector, Mendeley (http://www.mendeley.com/) and Papers (http://www.mekentosj.com/papers/), and in the public sector, CiteBank (http://citebank.org/).

Mendeley and Papers are both tools for an individual to organise their bibliographies. Both offer social network enhancements to enable papers to be shared among groups; though both restrict the number and size of groups and storage of references, that are available for free. If we were to work with either organisation then we will need to enter into a contractual relationship with them. Concerns over either organisation are their long term business plans and viability. The two named organisations represent the current leading on-line reference manager tools suitable for our use. There have been other earlier tools that rose, and then fell from prominence, such as CiteULike and Connotea. In a similar vein there is the publicly funded Zotero (http://www.zotero.org/), which has found a niche in the social sciences, but which would also require a commercial arrangement to handle the volumes of data a Bibliography of Life would generate.

Of the publicly funded bibliographic databases only CiteBank has the ambition to match the Bibliography of Life. Other databases are focused on a sub-domain of taxonomy and lack the scope to expand in line with the potential size of the Bibliography of Life. CiteBank is the bibliographic offshoot of the Biodiversity Heritage Library, which has achieved sustained funding (BHL:funding). However, its current vision is to continue as an index to BHL content only, and so is not suitable for building the Bibliography of Life that we envisage (Freeland, pers. comm.).

An alternative approach is not to build a Bibliography of Life database at all, but a functionally equivalent portal offering a federated search across existing taxonomic bibliographic resources. Hence, our task in ViBRANT would be to build a user interface to a global search of these existing data stores, complemented by an index to speed up query results. The latter would be necessary because we would have to do additional processing such as de-duplication on the fly to consolidate the results. The leading, proven indexing technology applicable to this task is Apache SOLR (http://lucene.apache.org/solr/). It offers many advantages if used in ViBRANT, not the least being its integration with Drupal, the foundation for Scratchpads. However, to build the index would still require that we address the same issues as if we were to populate our own reference database. Given the potential performance penalty, there seems to be no advantage in adopting a purely search portal approach over populating a searchable database.

Therefore, for performance reasons, and the ease with which we can offer additional benefits, we propose to build a database in place of a portal. Further, to ensure continuity of service, we will follow the lead set by DBLP and host the database within an academic institution. For the immediate delivery of the service we intend to host the Bibliography within our employing institution, the Open University. Longer term, we will explore the other hosting options made possible by the ViBRANT environment.

### Searching and extracting references

Having developed a database infrastructure, the second technical aspect to building a Bibliography of Life is extracting references from the database. For this we propose several approaches, including building our own dedicated search engine. However, we also intend to make use of existing services too, principally Mendeley.

There are several on-line tools for storing and sharing references. For the Bibliography of Life we intend to expose the references to Mendeley because it is the tool with the greatest coverage currently of taxonomic literature. This exposure will allow users to search the Bibliography of Life using a familiar tool, and should they wish, exploit the social networking aspects of Mendeley too. Note, the use of such tools is not without complications. For example, there are seven groups in Mendeley related to *ants* (Mendeley:ants), suggesting a fragmented approach to the researchers use of that tool.

These existing tools, however, do not deliver the full capability of a bibliography of Life. In particular, they will search primarily on published references and keywords. An advantage of hosting our own database is the extra value we can add by automatically reconciling author and journal names and extracting complementary metadata. Another possibility, if we can access the source document too, is for us to data mine it for additional keywords such as taxon names. These data can be added to the Bibliography of Life because we control its design, and we can provide a search engine to exploit this additional data.

### What it is not

The Bibliography of Life is not simply another search engine. Google (http://www.google.com/) is seemingly all-conquering in terms of popular search on the Internet. Its specialist academic derivative, Google Scholar (http://scholar.google.com/), is very popular too, based on informal, unscientific surveys. Yet these two search engines are not the solution to providing a Bibliography of Life.

Google and Google Scholar only search what is publicly available on the web. Private and personal bibliographies are not included in their results, neither in terms of breadth of coverage nor accuracy of information. These bibliographies are often a rich index to the pre-digital literature, which is not otherwise easily found even though the papers referenced are important in taxonomy. A Bibliography of Life can address this exposure, particularly for historic, taxonomic literature, which is only now being digitised and becoming publicly referenced on-line. Though it should be noted that contemporary, born-digital literature is well covered by these search engines.

A further complication arises from the different purpose of on-line search. For example, Google Scholar is aimed at helping researchers find articles, or related papers such as patent applications. Searches are based on authors or expected key words. If searching for keywords in the article itself, an overwhelming number of results can be returned. Defining a discriminating search query can be an arduous task. This could be made easier by the addition of appropriate metadata available to the search tool. A Bibliography of Life provides the opportunity to develop domain specific metadata to support searches. The relevance of the results is also affected by the granularity of the reference returned, especially when dealing with books or journal volumes. It would be far more productive to the taxonomist if the results referred directly to the relevant article, say, rather than the volume in which the article is found. This can be problematical in taxonomy, and other disciplines using scanned historic documents, because these are often indexed at the level of the scanned document rather than at the level of a meaningful search result (Page 2011a). The whole scanned document might not be the most appropriate level of reference.

Hence, we argue for the creation of specific taxonomic reference tool to assist the taxonomist locate and manage accurate references as being preferable to relying solely on generic search engines.

## Conclusion

This paper has outlined our intended approach to delivering a Bibliography of Life within the ViBRANT project. The Bibliography is specifically intended to benefit the professional and expert citizen scientist working in taxonomy. We have set out the social and technical issues that have prevented its creation before.

The social concerns focus on the willingness of users to contribute to the Bibliography. This can be addressed initially by automatically collating existing references. This will also allow us to begin exploiting these data for the benefit of our users, and enhancing the quality of the data. Sustainability will be achieved through making the Bibliography an integral part of a taxonomists’ workflow, and minimising any additional effort on their part to engage with it. We have shown how we intend to use Scratchpads to deliver this goal.

The technical concerns relate to the architecture required to deliver the Bibliography. We have argued that maximum benefit, in terms of being able to exploit the data, and greatest security of long term availability, is for us to build our own database. We recognise that users may wish to engage with the references using a variety of tools. We intend to expose the references to such new tools as Mendeley. In addition, to realise the maximum benefit from the data and the metadata we can extract from it, we will provide a dedicated search engine.

The ambitious vision of a comprehensive Bibliography of Life has not been realised before. In ViBRANT we have the commitment of a sufficiently large amount of time and resource to achieve a tool that can deliver more benefit to a taxonomist than existing smaller scale taxonomic bibliographic resources. In this, we will progress the vision of a “freely accessible bibliography of every taxonomic paper ever published” (Page 2010).

## References

[B1] Agosti D Egloff W (2009) Taxonomic information exchange and copyright: the Plazi approach. BMC Research Notes 2: 53. doi:10.1186/1756-0500-2-53. 10.1186/1756-0500-2-53 PMC2673227 19331688

[B2] BHL:funding http://biodivlib.wikispaces.com/Funding+Sources.

[B3] Feitelson DG (2004) On identifying name equivalences in digital libraries. Information Research 9(4): 192.

[B4] Gupta D Morris B Catapano T Sautter G (2009) A new approach towards bibliographic reference identification, parsing and inline citation matching. In: Proceedings of the International Conference on Contemporary Computing, Noida (India), August 2009.

[B5] Hall D (2010) How many journal articles have been published (ever)? http://duncan.hull.name/2010/07/15/fifty-million/

[B6] Hargreaves I (2011) Digital Opportunity: A review of Intellectual Property and Growth. Crown copyright. http://www.ipo.gov.uk/ipreview.htm?intcmp=239

[B7] Kan M-Y Tan YF (2008) Record matching in digital library metadata. Communications of the ACM 51: 91-94. 10.1145/1314215.1340938

[B8] Lee D Kang J Mitra P Giles CL On B-W (2007) Are your citations clean? Communications of the ACM 50: 33–38. 10.1145/1323688.1323690

[B9] Ley M (2009) DBLP - Some Lessons Learned. In: Proceedings of the Very Large Databases Conference, Lyon (France), August 2009.

[B10] Ley M Reuther P (2006) Maintaining an online bibliographical database: The problem of data quality. In: Actes des sixièmes journées Extraction et Gestion des Connaissances, Lille (France), January 2006.

[B11] McKay D Sanchez S Parker R (2010) What’s My Name Again? Sociotechnical Considerations for Author Name Management in Research Database. In: Proceedings of the 22nd Conference of the Computer-Human Interaction Special Interest Group of Australia on Computer-Human Interaction - OZCHI ’10, Brisbane (Australia), November 2010. doi:10.1145/1952222.1952274.

[B12] Mendeley: ants http://www.mendeley.com/groups/search/?query=ants.

[B13] Mendeley: reference extraction http://feedback.mendeley.com/forums/4941-mendeley-feedback/suggestions/834313-version-0-9-7-does-not-extract-references-from-the-Mendeley.

[B14] Page R (2010) Mendeley, BHL, and the “Bibliography of Life”. http://iphylo.blogspot.com/2010/10/mendeley-bhl-and-of-life.html

[B15] Page R (2011a) Extracting scientific articles from a large digital archive: BioStor and the Biodiversity Heritage Library. BMC Bioinformatics 12: 187. 10.1186/1471-2105-12-187 PMC3129327 21605356

[B16] Page R (2011b) Microcitations: linking nomenclators to BHL. http://iphylo.blogspot.com/2011/03/microcitations-linking-nomenclators-to.html

[B17] Phua C, Lee V Smith K (2006) The Personal Name Problem and a Recommended Data Mining Solution. In: Wang J (Ed) Encyclopedia of Data Warehousing and Mining. Idea Group, London.

[B18] Plagiarism Today (2011)http://www.plagiarismtoday.com/2011/08/08/using-citations-to-detect-plagiarism/

[B19] Redman TC (1996) Data Quality for the Information Age. Artech House, London.

[B20] Rinaldo C, Norton CN (2010) The Biodiversity Heritage Library: an expanding international collaboration. In: Proceedings of the 36th International Association of Aquatic and Marine Science Libraries and Information Centers Conference, Mar del Plata (Argentina), October 2010.

[B21] Sautter G Böhm K Agosti D (2007) Semi-automated XML markup of biosystematic legacy literature with the GoldenGATE editor. In: Pacific Symposium on Biocomputing 2007, Maui, Hawaii (USA), January 2007. 17992751

[B22] Time: Gaddafi (2011)http://newsfeed.time.com/2011/02/23/how-do-you-spell-gaddafi-the-linguistics-behind-libyas-leader/

[B23] W3C: Internationalisation http://www.w3.org/International/.

[B24] W3C: Personal names http://www.w3.org/International/questions/qa-personal-names.

[B25] Yahoo: Gaddafi (2011)http://uk.news.yahoo.com/how-should-you-spell-gaddafi%E2%80%99s-name-.html

